# Polymer-Based Scaffolds Incorporating Selected Essential Oil Components for Wound Healing: A Review

**DOI:** 10.3390/pharmaceutics17101313

**Published:** 2025-10-09

**Authors:** Vuyolwethu Khwaza, Opeoluwa O. Oyedeji

**Affiliations:** Department of Chemical and Earth Sciences, Faculty of Science and Agriculture, University of Fort Hare, Alice 5700, South Africa

**Keywords:** essential oils, polymers, wound dressings, wound healing, nanofibers, hydrogels, films

## Abstract

**Background:** The treatment of wounds remains a significant clinical challenge, particularly in chronic and infected wounds, where delayed healing often results in complications. Recent advances in biomaterials have highlighted the potential of polymer-based scaffolds as promising platforms for wound management due to their ability to mimic the extracellular matrix, support tissue regeneration, and provide a moist environment conducive to healing. **Objectives:** This review aims to provide a comprehensive overview of the recent progress in the design and application of polymer-based scaffolds loaded with essential oil (EO) components, emphasizing their role in promoting effective wound healing. **Methods:** Relevant literature on polymeric scaffolds and EO-based bioactive agents was systematically reviewed, focusing on studies that investigated the biological activities, fabrication techniques, and therapeutic performance of EO-loaded scaffolds in wound management. **Results:** Findings from recent studies indicate that EO components, particularly monoterpenoids such as thymol, carvacrol, and eugenol, exhibit remarkable antimicrobial, anti-inflammatory, antioxidant, and analgesic properties that accelerate wound healing. When incorporated into polymer matrices, these components enhance scaffold biocompatibility, antimicrobial efficacy, and tissue regeneration capacity through synergistic interactions. **Conclusions:** The integration of essential oil components into polymeric scaffolds represents a promising strategy for developing multifunctional wound dressings. Such systems combine the structural advantages of polymers with the therapeutic benefits of EOs, offering an effective platform for accelerating healing and preventing wound infections.

## 1. Introduction

A wound can be defined as a disruption of the skin’s structure, an injury that results in the loss of continuity of the skin, mucous membranes, or underlying tissues. Such damage may arise from external causes, including physical, chemical, electrical, thermal, or pressure-related factors, as well as from medical conditions or physiological disorders such as diabetes and malignancies. Wound healing is a complex and dynamic biological process that involves multiple overlapping stages, including hemostasis, inflammation, proliferation, and tissue remodeling [1,2]. In clinical settings, impaired wound healing is a major concern, particularly in the case of chronic wounds, diabetic ulcers, and infections, which can result in prolonged recovery, increased healthcare costs, and decreased quality of life [3]. In the United States, chronic wounds affect around 6.5 million people, with an estimated annual cost of approximately 25 billion dollars dedicated to wound management annually [4]. To address these challenges, the development of advanced wound dressings that promote rapid and effective healing has become a critical area of research.

Polymeric scaffolds have emerged as one of the most promising materials in wound care, owing to their biocompatibility, structural similarity to the extracellular matrix (ECM), and ability to support cell adhesion, proliferation, and tissue regeneration. Both natural (e.g., chitosan, gelatin, alginate) and synthetic (e.g., polycaprolactone, polylactic acid) polymers are widely utilized to fabricate scaffolds with tailored properties for wound management. Recently, there has been growing interest in incorporating bioactive agents into polymeric scaffolds to enhance their therapeutic efficacy. Among these, EO components, particularly phenolic monoterpenoids and related compounds such as carvacrol (CAR), thymol (THY), and eugenol (EUG) have demonstrated significant antibacterial [5,6], anti-inflammatory [7,8], antioxidant [9,10,11], and wound-healing [12,13] properties. Their integration into polymeric scaffolds provides a synergistic approach, combining the mechanical and structural support of the polymer matrix with the biological activity of EO components.

**Figure 1:**
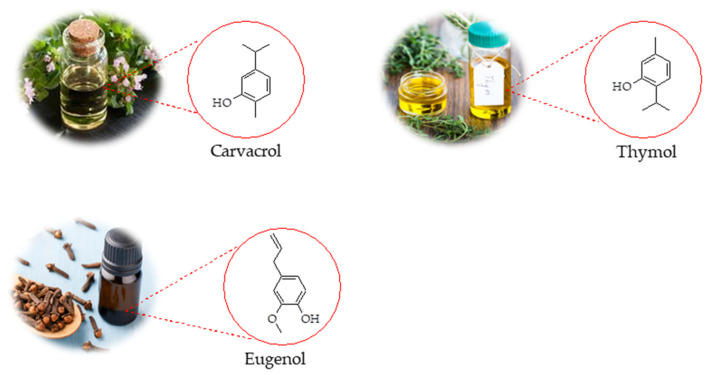
Chemical structures of selected EO components extracted from different plant species.

This review aims to provide a comprehensive overview of recent progress in the development of polymer-based scaffolds incorporating selected EO components such as CAR, THY, and EUG (in Figure 1) for wound treatment. It highlights the mechanisms of wound healing, properties of selected EO components relevant to wound therapy, types of polymers used, fabrication techniques, and therapeutic outcomes.

### Scope of the Literature Search

To define the scope of this review, we conducted a targeted literature search using databases such as Google Scholar, PubMed, Scopus, and Web of Science. The search focused on publications using keywords including polymer-based scaffolds, essential oils, wound healing, antibacterial, anti-inflammatory and the names of specific EO components such as carvacrol, thymol, and eugenol. The search yielded 170 primary research articles, of which approximately 38 (from 2014–2025) specifically investigated the polymer-based scaffolds incorporating selected EO components for wound healing. While there are several general reviews on EOs or wound healing polymers, only a limited number (~4–7) of reviews comprehensively address their integration [14,15,16,17]. This review aims to bridge that gap by critically examining the therapeutic potential and application of selected EO components within polymer-based scaffolds for wound care.

## 2. EOs and Their Active Constituents in Wound Healing

Plants are capable of producing two types of oils: fixed oils and EO. Fixed oils, also referred to as triacylglycerols or triglycerides, are composed of a glycerol backbone bonded to three fatty acid chains. In contrast, EOs, also known as volatile oils, essences, etheric oils, or aetheroleum, are complex natural mixtures of aromatic, volatile, and lipophilic compounds typically found in aromatic plants. Most EOs are clear or light yellow, remain in liquid form at room temperature, and are generally less dense than water, with a few exceptions such as oils from cinnamon, sassafras, and vetiver [18]. EOs and their components derived from aromatic plants have garnered significant interest due to their wide range of biological activities, particularly their strong antimicrobial properties [19,20]. During the wound healing process, essential oils can promote faster wound closure, enhance collagen formation, and stimulate fibroblast proliferation [14]. These components consist of complex mixtures of bioactive molecules, with phenolic monoterpenoids such as CAR, THY, and EUG etc., playing a central role in their pharmacological activities [21]. Due to their distinctive flavors, fragrances, and therapeutic effects, these compounds are utilized across a wide range of sectors, including agriculture [22], cosmetics [23,24], pharmaceuticals [25], and food packaging [26]. These EO components have been shown to exhibit a wide range of pharmacological activities, including antibacterial [27], antioxidant [9,28], and anti-inflammatory effects [29,30].

Several studies have demonstrated that the selected EO components significantly accelerate the wound healing process [13,31,32]. Based on the findings of the study by Amirzade-Iranaq et al., the application of *Mentha piperita* (peppermint) EO enhanced the expression of the TGF-β gene in the wound site, contributing to wound healing by promoting the release of growth factors and creating a favorable environment for tissue repair. The EO exhibits antibacterial properties due to the presence of compounds such as THY, phenols, flavonoids, and oxidant-rich terpenes. Furthermore, the antioxidant activity of these constituents supports angiogenesis, stimulates fibroblast proliferation and epithelialization, helps control skin infections, and accelerates the overall wound healing process [33]. Another study conducted by Modarresi et al. demonstrated that the topical application of *M. piperita*, particularly at higher concentrations, significantly enhanced fibroblast numbers, collagen regeneration, and epithelialization in the wound area on days 7 and 14. However, molecular analysis showed a reduction in the expression of genes associated with angiogenesis (VEGF) and fibroblast recruitment (FGF-2) in the *M. piperita*-treated groups compared to the negative control group [34].

The systematic review by Costa et al. highlighted the significant role of CAR and THY in all three phases of wound healing. During the initial phase, these compounds exhibited anti-inflammatory, antioxidant, and antimicrobial activities by modulating inflammatory cytokines and oxidative stress. In the proliferative phase, they supported re-epithelialization, angiogenesis, and the formation of granulation tissue. In the final remodeling phase, CAR and THY enhanced collagen deposition and regulated the proliferation of fibroblasts and keratinocytes, thereby contributing to effective tissue repair [31]. Gunal et al. investigated the effects of CAR on wound healing following excisional skin injury. During the early phase of healing, CAR treatment led to enhanced granulation tissue formation and a moderate reduction in wound depth, which was linked to increased TNF-α levels. In the later stages of the study, a significant rise in TGF-β1 levels was observed in the CAR-treated group compared to the controls. Additionally, IL-1β levels were elevated only on day 8 in the treated animals. These findings suggest that CAR promotes wound healing by modulating key pro-inflammatory mediators, including TNF-α, IL-1β, and TGF-β1 [35]. Tabatabaein et al. examined the molecular effects of CAR on human dermal fibroblasts (HDFs) cultured under high-glucose conditions. CAR treatment enhanced collagen deposition and promoted wound closure in the cells. At a concentration of 9 µM, there was an upregulation of TGFβ1 and ACTA2 mRNA levels, along with a reduction in miR-155 expression. In addition to its known antimicrobial activity, CAR demonstrated anti-inflammatory, pro-proliferative, and tissue remodeling properties, highlighting its potential role in wound healing [36].

The antimicrobial activity of THY and CAR is partly due to their disruption of microbial plasma membranes, causing increased permeability and leakage of cellular contents. This effect depends on the drugs’ physicochemical properties (like lipophilicity and solubility) as well as the lipid makeup and surface charge of the microbial membranes. Additionally, these compounds may penetrate the cells and interact with internal targets essential for antibacterial function [37,38].

## 3. Wound Healing: Physiology and Phases

### 3.1. Phases of Wound Healing

**Figure 2:**
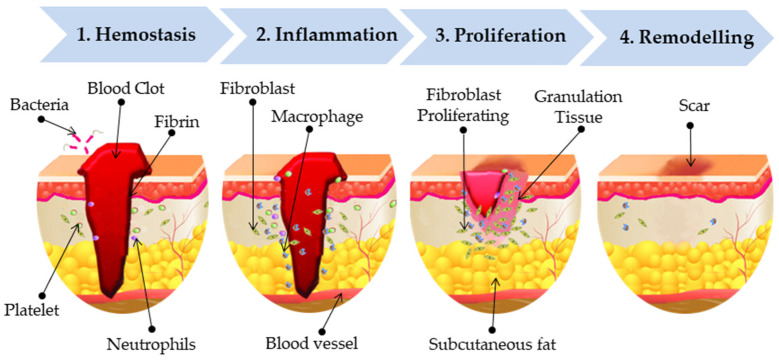
Sequential wound healing phases.

The skin, the body’s largest organ, regulates temperature, maintains homeostasis, serves as a sensory organ, and acts as a barrier against microbes. This protective barrier is compromised when the skin is damaged [39]. If wounds are not properly treated, they can lead to complications such as bleeding, inflammation, infection, scarring, impaired angiogenesis and tissue regeneration [40]. Skin repair is a highly complex physiological process that depends on the precise coordination of multiple cell types, along with chemokines, cytokines, and diverse growth factors acting in a sequential manner [40]. Wounds are generally classified into two main types based on their healing duration: acute wounds and chronic wounds [41]. Acute wounds are sudden injuries, such as cuts or surgical incisions that heal in a predictable and timely manner, usually within 2–3 weeks [42]. Chronic wounds, on the other hand, fail to follow the normal healing process, persist for longer periods, and are often linked to underlying conditions like diabetes or vascular disorders [43,44]. Wound healing phases occur through three interconnected yet distinct biological phases: hemostasis and inflammation, followed by proliferation, and finally tissue remodeling (Figure 2) [45]: 1. Hemostasis: This is the immediate response after injury, where platelets aggregate to form a blood clot stabilized by fibrins. This clot not only stops bleeding but also acts as a temporary barrier against bacteria, while neutrophils migrate to the wound site as part of the initial immune defense [46,47]. 2. Inflammation: During this phase, immune cells such as macrophages and neutrophils clear debris, pathogens, and damaged tissue. At the same time, fibroblasts are recruited, and nearby blood vessels dilate, allowing more immune cells to enter the wound area [48,49]. 3. Proliferation: Here, fibroblasts proliferating play a key role in synthesizing extracellular matrix and collagen, which form the basis of granulation tissue [48]. This tissue fills the wound bed and supports angiogenesis and re-epithelialization [41,45,50]. 4. Remodelling (Maturation): In the final stage, connective tissue develops alongside replacement epithelium, leading to the formation of scar tissue [51]. Collagen fibers are realigned and strengthened, restoring skin integrity and increasing the tensile strength of the healed tissue [52,53,54].

### 3.2. Factors Affecting the Wound Healing Process

**Figure 3:**
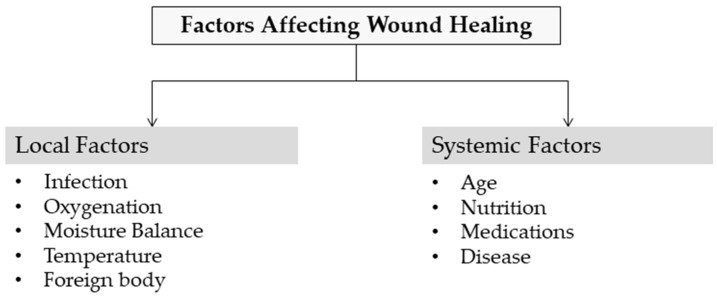
Factors affecting wound healing.

Several factors contribute to impaired wound healing and can generally be divided into local and systemic categories (Figure 3). Local factors directly impact the wound site itself, while systemic factors relate to the individual’s overall health or disease condition, which in turn influence healing through local effects. These factors are often interconnected, with systemic conditions exacerbating local wound responses [55]. Identifying these factors and making the correct diagnosis are the first steps in treating chronic wounds.

#### 3.2.1. Local Factors

Infection: When the skin is injured, microorganisms residing on its surface can penetrate into the underlying tissues. The extent of their growth and infection determines whether the wound is classified as contaminated, colonized, locally infected/critically colonized, or as a spreading invasive infection [56]. Contamination describes the presence of non-replicating microorganisms on a wound, whereas colonization refers to replicating microbes that do not necessarily cause tissue injury. Local infection or critical colonization represents an intermediate stage where microbial replication begins to elicit localized tissue responses. Invasive infection occurs when replicating microorganisms penetrate the wound and cause significant host tissue damage [55]. Inflammation is a natural and essential phase of wound healing, playing a key role in eliminating invading microorganisms. However, if decontamination is ineffective, inflammation becomes prolonged due to incomplete microbial clearance. Both bacteria and their endotoxins can sustain high levels of pro-inflammatory cytokines such as IL-1 and TNF-α, extending the inflammatory phase. Prolonged inflammation may push the wound into a chronic state, preventing proper healing. This condition is also characterized by elevated levels of matrix metalloproteinases (MMPs), which degrade the extracellular matrix (ECM), along with reduced levels of natural protease inhibitors. Such an imbalance accelerates the breakdown of growth factors present in chronic wounds, further impairing repair [57].

Oxygenation: The importance of oxygen in wound healing has been recognized since the 1960s, when studies showed that adequate oxygen supply promotes granulation tissue formation and collagen synthesis. Oxygen is vital for generating ATP and other energy sources through cellular respiration. During wound repair, sufficient oxygenation supports key processes such as cell proliferation, antimicrobial defense, angiogenesis, collagen production, and epithelialization. These functions are crucial to meet the heightened energy demands of tissue regeneration and ensure effective healing [58]. Various systemic conditions, including aging and diabetes, can restrict blood circulation, leading to insufficient oxygen supply in tissues and the development of hypoxic wounds. Chronic wounds are often severely hypoxic, and healing is impaired unless oxygenation is restored. While short-term hypoxia after injury can initiate the healing process, prolonged or persistent hypoxia delays wound repair [59].

Moisture Balance: Maintaining proper moisture balance is a critical factor in regulating the wound’s microenvironment. Wound-healing approaches encompass traditional dry healing, moist healing, and the more advanced concept of achieving an optimal moisture balance [60]. The conventional dry healing approach assumes that wounds heal best in a dry environment with the presence of oxygen. However, in reality, the oxygen that supports wound repair is supplied through hemoglobin in the blood, while atmospheric oxygen cannot be directly utilized by the wound tissue [61]. Due to an unfavorable healing environment, dry healing often leads to wound dehydration and scab formation, which hinder epithelial cell migration. It also results in the loss of bioactive substances, ultimately delaying the overall healing process [62,63]. The moist wound healing theory emphasizes that maintaining a moist environment helps regulate oxygen tension within the wound and stimulates capillary formation, thereby accelerating the healing process. In addition, moisture preserves proteolytic enzymes present in the wound exudate, which aid in the breakdown and clearance of necrotic tissue [61]. Nevertheless, growing evidence indicates that wound exudate provides a favorable medium for bacterial growth, making the wound highly susceptible to infections [64]. Furthermore, excessive moisture at the wound site may cause maceration, edema, and dysfunction of wound-edge cells in the surrounding healthy tissue, thereby interfering with the normal healing process. This has led to growing interest in the moisture balance theory among researchers [60]. The moisture balance theory highlights that maintaining an optimal level of humidity creates a favorable microenvironment for wound repair, supporting cell migration, proliferation, and overall tissue regeneration [65,66]. Wound dressings play a vital role in establishing and regulating a moist environment that supports healing. These moist dressings are commonly categorized into films, foams, hydrocolloids, hydrogels, and alginates [67].

Temperature: Wound temperature is a key indicator of the local microenvironment and has been suggested as a potential predictor of infection and healing progress. However, further research is needed to validate its clinical relevance. A clearer understanding of how wound temperature correlates with healing could provide valuable insights for clinical management and support the design of advanced smart dressings. Huang et al. reported that applying localized heat to hypothermic wounds enhances capillary perfusion and oxygen availability, stimulates neutrophil, fibroblast, and keratinocyte activity with increased collagen deposition, and raises lymphocyte levels. These effects collectively boost the innate immune response and promote wound repair [68]. Derwin et al. reviewed the influence of topical agents and dressings on wound surface pH, temperature, and healing. A clinical study showed that topical sodium nitrite raised peri-wound temperature and reduced leg ulcer size; however, due to limited evidence, no specific topical agent or dressing can yet be recommended for manipulating pH or temperature to enhance wound healing outcomes [69]. Lin et al. [70] investigated temperature differences between the wound bed, periwound skin, and normal skin using infrared thermometry. They observed that higher periwound skin temperature correlated with improved healing, whereas lower periwound temperature was linked to latent necrosis, consistent with findings by Kanazawa et al. [71]. Similarly, Nakagami et al. reported that delayed healing occurs when wound-bed temperature exceeds periwound temperature, likely due to disruptions in the normal distribution of metabolic heat through blood flow and perfusion [72]. Overall, wound temperature represents a promising biomarker for monitoring infection risk and predicting healing outcomes. Evidence suggests that both localized heating and temperature differentials between the wound bed and periwound skin can influence cellular activity, immune response, and tissue repair. However, current findings remain limited, and further clinical studies are required to establish standardized temperature-based strategies and guide the development of smart dressings for optimized wound management.

Foreign body: Foreign bodies within a wound frequently cause delayed healing or may result in the development of a draining sinus or tract. Their presence triggers a sustained inflammatory response that interferes with normal repair. In veterinary cases, common examples include grass seeds, bone fragments, teeth, and sticks introduced through puncture injuries. Surgical implants act as foreign bodies, and improper selection of sutures or drains can complicate wound healing. Drains, while useful for reducing dead space and fluid accumulation, may weaken local resistance to infection and provide entry points for bacteria. Therefore, their use should be carefully justified, with strict aseptic technique and minimal duration to reduce complications [73]. When choosing suture material, the smallest gauge and the minimal amount required for wound closure should be used to limit the introduction of foreign material. In traumatic wounds, multifilament sutures should be avoided since their fiber interstices can harbor bacteria, which immune cells such as neutrophils and macrophages cannot effectively penetrate [73]. Foreign bodies, including surgical implants, sutures, and drains, can significantly hinder wound healing by sustaining inflammation and increasing infection risk. Careful selection of materials, adherence to aseptic techniques, and minimizing foreign body load are essential strategies to reduce complications and support effective tissue repair.

#### 3.2.2. Systemic Factors

Age: Wound healing in older individuals is often slower compared to younger patients, largely due to age-related comorbidities. Factors such as hormonal imbalances, dehydration, and declines in immune, circulatory, and respiratory function contribute to a higher risk of skin breakdown and delayed repair [74]. In elderly individuals, monocytes exhibit an overexpression of cytokines, particularly pro-inflammatory cytokines and interleukin-6 (IL-6), which leads to a prolonged initial inflammatory phase and delayed macrophage infiltration at the injury site [75]. Similar findings have been observed in aged mice, which demonstrate slower recovery with less differentiated fibroblasts and myofibroblasts [76]. In fibroblasts, this delay is largely attributed to reduced transforming growth factor-β (TGF-β) expression and diminished sensitivity to TGF-β–mediated signaling pathways [76]. Additionally, in older individuals, the ratio of soluble IL-6 Rα decreases, further weakening IL-6/IL-6 Rα-mediated stimulation of gp130 in endothelial cells, keratinocytes, fibroblasts, and macrophages [77]. Consequently, this impairs the transition of macrophages from the M1 phenotype to the pro-proliferative M2 phenotype [78]. Overall, aging significantly slows wound healing due to multiple physiological and cellular alterations. Elevated IL-6 levels, reduced TGF-β activity, impaired macrophage transition from M1 to M2 phenotype, and diminished fibroblast function collectively prolong inflammation and limit tissue repair. These age-related changes increase the risk of chronic wounds, impaired proliferation, and delayed scarring in older individuals.

Nutrition: Previous studies have shown that nutrition plays a critical role in the wound-healing process [79]. Hunger or dietary deficiencies following trauma or surgical procedures can significantly impair tissue repair. Patients with chronic dietary deficiencies and non-healing wounds often require specific nutritional supplementation. The healing outcome is strongly influenced by the metabolism of calories, carbohydrates, minerals, lipids, vitamins, and proteins [55,75]. Since the cells involved in wound healing depend on proteins for their development and function, protein loss can adversely impact the entire immune process. Proteins are essential for an effective immune response, and any impairment may hinder the transition from the inflammatory to the proliferative phase. Furthermore, during the proliferative and remodeling stages, protein-energy deficiency can suppress fibroblast activity, leading to delayed angiogenesis and reduced collagen synthesis [80]. Beyond proteins, both carbohydrates and fats play critical roles in meeting the increased energy demands required to sustain the inflammatory response, cellular activity, angiogenesis, and collagen deposition during the proliferative phase of wound healing [81]. Adequate carbohydrate intake is particularly important for fibroblast proliferation and migration, as well as for leukocyte function [82]. Carbohydrates also stimulate the release of hormones and growth factors, such as insulin, which are essential for the anabolic processes of the proliferative phase. Conversely, hyperglycemia and its associated complications can impair granulocyte function and increase susceptibility to wound formation [83]. Fats, on the other hand, serve structural roles in the lipid bilayer of cell membranes during tissue regeneration. They also act as precursors of prostaglandins, which function as mediators of cellular inflammation and metabolism, and are involved in various signaling pathways. However, the role of essential fatty acid supplementation in wound healing remains controversial. For instance, omega-3 supplementation has been reported to decrease wound tensile strength, negatively impacting healing, while a combination with omega-6 has been shown to slow the progression of pressure ulcers. Accordingly, co-supplementation with omega-3 and omega-6 may provide therapeutic benefits, particularly during the inflammatory phase [79].

Medications: Currently, several approved drugs are known to negatively affect wound healing by disrupting the coagulation cascade, inflammatory processes, or cellular proliferation [55]. Corticosteroids, commonly prescribed as anti-inflammatory and immunomodulatory agents, impair healing by reducing the expression of growth factors and cytokines, thereby interfering with multiple regulatory pathways of tissue repair, and by suppressing fibroblast proliferation [80]. Similarly, non-steroidal anti-inflammatory drugs (NSAIDs), when used systemically to manage pain and inflammation, can hinder healing by decreasing fibroblast proliferation, slowing wound contraction, and delaying angiogenesis. In contrast, topical NSAID formulations have been shown to enhance wound healing while alleviating local pain [84]. Chemotherapeutic agents also present challenges for wound repair, as their mechanisms of action inhibit cellular metabolism and proliferation, ultimately resulting in impaired re-epithelialization, reduced angiogenesis, diminished collagen synthesis, and delayed wound contraction [80].

Diseases: Coronary artery disease, peripheral vascular disease, cancer, and diabetes mellitus are a few of the chronic diseases that can compromise wound healing by disrupting key mechanisms such as inflammation, angiogenesis, re-epithelialization, and extracellular matrix remodeling. Diabetes mellitus is a well-documented example, as it is a multifactorial condition that profoundly impairs healing. It interferes with leukocyte migration and activation, enhances the release of pro-inflammatory cytokines, and contributes to a state of persistent inflammation [85]. In addition, diabetes compromises the skin’s microvasculature, creating a hypoxic environment that reduces angiogenesis, while also impairing keratinocyte and fibroblast proliferation and differentiation—ultimately delaying re-epithelialization and matrix remodeling [86]. Obesity is another chronic condition linked with impaired wound healing, often associated with complications such as pressure ulcers and venous ulcers, frequently accompanied by hematoma, edema, seroma formation, and local infection [87,88]. At the cellular and molecular levels, delayed wound healing in obesity is driven by diminished skin microperfusion, excessive secretion of pro-inflammatory cytokines, and reduced immune competence [55,87].

## 4. Biological Properties of Selected EOs Components Relevant to Wound Healing

The selected EO components exhibit a broad spectrum of biological activities such as anti-inflammatory and antimicrobial activities that directly address the key physiological processes involved in wound repair. Research suggests that THY, CAR, and EUG exert their biological activities through multiple mechanisms of action. The following sections discuss the anti-inflammatory and antibacterial activities of THY, CAR, and EUG.

### 4.1. Anti-Inflammatory Activity

Inflammation is a complex and dynamic process of the innate immune system that helps defend and protect the body when homeostasis is disrupted by chemical, mechanical, or biological factors. Clinically, it is characterized by heat, redness, swelling, pain, and loss of tissue function. This response involves tightly regulated molecular and cellular mechanisms that can have both local and systemic effects, ultimately aiming to repair tissue and restore balance in the body [89,90]. The inflammatory process often triggers the production of reactive oxygen species (ROS), which in turn contribute to oxidative stress. These highly reactive molecules, when generated in excess, can surpass the body’s antioxidant defense capacity, leading to cellular injury and disruption of normal homeostasis. Inflammation-induced oxidative stress has been linked to the development of several diseases, such as cardiovascular disorders, neurodegenerative conditions, and certain cancers [91,92]. Thus, controlling it is essential for health.

This process is driven by complex molecular mechanisms that engage multiple signaling pathways, leading to the release of inflammatory mediators and the recruitment of immune cells. Gaining a deeper understanding of these pathways is vital for the discovery of novel therapeutic targets and the development of new drugs [93]. The nuclear factor-kappa B (NF-κB) signaling pathway plays a central role in regulating the inflammatory response by promoting the transcription of pro-inflammatory genes, such as tumor necrosis factor-α (TNF-α), interleukin-1β (IL-1β), interleukin-6 (IL-6), and nitric oxide (NO). Clinically, NSAIDs and glucocorticoids are commonly used to manage inflammatory conditions [94]. However, their use is often limited by adverse effects including gastrointestinal ulcers, bleeding, constipation, and abdominal pain and suboptimal therapeutic outcomes [11,95]. Consequently, the search for new bioactive compounds with improved safety and efficacy profiles is of critical importance. EO components such as THY, CAR, and EUG are among the most extensively studied natural polyphenolic compounds, owing to their broad spectrum of biological activities. Evidence from multiple studies confirms that the selected EO components exhibit anti-inflammatory properties [11,96,97,98,99,100,101].

#### 4.1.1. Carvacrol/Thymol Anti-Inflammatory

Carvacrol (5-isopropyl-2-methylphenol, Figure 1) is a monoterpenoid and an isomer of thymol(2-isopropyl-5-methylphenol, Figure 1), both belonging to the *p*-menthane class. They are biosynthesized from geranyl pyrophosphate (GPP) as the precursor, with γ-terpinene serving as an intermediate [102]. These compounds are commonly present in the EOs of numerous aromatic plants [89]. Numerous review articles have highlighted the anti-inflammatory and wound-healing properties of CAR and THY [31,98]. These diverse properties, along with others not discussed here, underscore their potential as valuable natural compounds for pharmaceutical and cosmetic applications.

Inflammation plays a central role in the development of various chronic diseases and can be regulated by targeting critical molecular pathways, including MAPK, NF-κB, JAK/STAT, and arachidonic acid signaling. THY has been shown to modulate these pathways, leading to a reduction in the production of pro-inflammatory cytokines and mediators [7]. CAR has demonstrated potential in the treatment of inflammatory skin conditions, as it effectively targets two key aspects of inflammation, edema formation and leukocyte infiltration, and appears to contribute to the acceleration of the healing process. These findings support the traditional use of CAR-rich plants for their anti-inflammatory properties [103]. Fachini-Queiroz et al. investigated the anti-inflammatory effects of *Thymus vulgaris* EO and its major constituents, THY and CAR, using experimental models including ear edema, carrageenan-induced pleurisy, and in vitro chemotaxis. In the pleurisy model, *Thymus vulgaris* EO, CAR, and THY significantly reduced inflammatory edema, although only *Thymus vulgaris* EO and CAR inhibited leukocyte migration. In the chemotaxis assay, CAR suppressed leukocyte migration, while THY acted as a strong chemoattractant. In the ear edema model, topical application of CAR (10 mg/ear) effectively reduced edema, indicating a local anti-inflammatory effect. In contrast, THY did not reduce edema and instead induced an irritant response, likely mediated by histamine and prostanoid release [29]. The anti-inflammatory and uterine relaxant effects of THY and CAR, likely mediated through calcium channel blockade, suggest their potential as effective and safe tocolytic agents, offering promising improvements to current pharmacological strategies for managing preterm labor [104]. Findings by Gholijan et al. demonstrated that CAR significantly reduced IL-1β and TNF-α expression at both the protein and mRNA levels, while THY significantly suppressed IL-1β expression. Western blot analysis of nuclear extracts showed that both compounds markedly decreased the expression of c-Fos, NFAT-1, and NFAT-2, with c-Jun downregulation observed only in response to CAR. Neither compound affected the expression of phosphorylated NF-κB p65. At the protein level, both CAR and THY reduced levels of inducible phospho-SAPK/JNK and phospho-STAT3; however, only CAR increased phosphorylated p38 levels in total cell extracts. Notably, although both agents reduced phospho-IκBα levels, phosphorylated NF-κB p65 remained elevated in the presence of CAR [105]. The combination of THY and CAR exhibited strong anti-inflammatory activity, significantly suppressing the expression of 5-lipoxygenase (5-LOX), cyclooxygenase-1 (COX-1), and cyclooxygenase-2 (COX-2) enzymes [106]. This exhibited a synergistic cardioprotective effect, attributable to their combined antioxidant, anti-inflammatory, and anti-apoptotic properties [107]. CAR/THY derivatives demonstrated anti-inflammatory activity at a concentration of 10 μM by inhibiting LPS-induced IL-1β release in BV2 microglial cells. Among the tested compounds, one exhibited a notable IC_50_ value of 8.33 ± 0.08 μM. Additionally, this compound significantly reduced IL-1β levels in an Alzheimer’s disease mouse model, indicating strong in vivo anti-inflammatory efficacy. In behavioral assessments, it markedly improved scopolamine-induced memory impairment, restoring performance to near-normal levels, evidenced by reduced latency, more direct navigation, and increased platform crossings in the Morris water maze test [108].

CAR and THY exhibit notable anti-inflammatory activity through various molecular mechanisms (see Table 1). CAR consistently demonstrates broader and more potent effects compared to THY, including inhibition of edema formation, leukocyte infiltration, and downregulation of key inflammatory mediators such as IL-1β and TNF-α at both transcriptional and protein levels. It also modulates signaling pathways including MAPK, STAT3, and NFAT, with selective activation of phospho-p38. THY, while effective in reducing IL-1β expression and modulating some signaling proteins, shows a less comprehensive anti-inflammatory profile and even provokes irritant responses in some models. Combined administration of CAR and THY yields synergistic effects, significantly suppressing COX-1, COX-2, and 5-LOX expression, and offering cardioprotective and neuroprotective benefits. Derivatives of these compounds have also demonstrated promising in vivo efficacy, particularly in neuroinflammation and cognitive function restoration, highlighting their therapeutic potential in both inflammatory and neurodegenerative disorders.

#### 4.1.2. Eugenol Anti-Inflammatory

Eugenol (4-allyl-2-methoxyphenol, Figure 1) is a phenylpropanoid compound commonly found in the EOs of various plant families, including Lamiaceae, Lauraceae, Myrtaceae, and Myristicaceae [109]. In recent years, growing evidence has highlighted eugenol’s anti-inflammatory, analgesic, and antioxidant properties, supporting its potential therapeutic applications in cancer, inflammatory bowel disease, renal and pulmonary injuries, and osteoarthritis [11].

EUG has demonstrated significant therapeutic potential in models of inflammation and oxidative stress. In lipopolysaccharide (LPS)-induced acute lung injury (ALI), eugenol administration led to a dose-dependent improvement in both pulmonary function and histological structure. At a dosage of 150 mg/kg, it effectively suppressed the release of pro-inflammatory cytokines (TNF-α, IL-1β, and IL-6), inhibited NADPH oxidase activity, and modulated the activity of key antioxidant enzymes, including superoxide dismutase, catalase, and glutathione peroxidase. Additionally, EUG reduced protein oxidation associated with LPS exposure, indicating its combined anti-inflammatory and antioxidant mechanisms that preserve lung architecture [110]. In other inflammatory contexts, both cinnamaldehyde and EUG inhibited the secretion of pro-inflammatory cytokines from peripheral blood mononuclear cells (PBMCs) derived from rheumatoid arthritis (RA) patients. These compounds also attenuated the formation of reactive oxygen and nitrogen species, contributing to decreased biomolecular oxidation and enhancement of antioxidant defenses. These findings suggest their potential use as adjunct therapies in RA due to their dual free radical scavenging and anti-inflammatory actions [111]. Molecular docking studies further supported EUG’s therapeutic promise, revealing that both EUG and its derivative, acetyleugenol, possess favorable binding affinities to the COX-2 enzyme, a key player in inflammation. In silico ADMET (absorption, distribution, metabolism, excretion, and toxicity) analyses predicted that both compounds have acceptable safety profiles, showing no evidence of toxicity or carcinogenicity. Consequently, EUG was extracted from *Eugenia caryophyllata* essential oil and chemically modified to acetyleugenol for in vivo experimentation, where both compounds exhibited notable anti-inflammatory effects, underscoring their value as lead candidates for anti-inflammatory drug development [112]. A review by Devu et al. highlighted eugenol’s potential in cardiovascular disease prevention through its antioxidant activity, particularly its capacity to scavenge free radicals involved in inflammation and tissue injury. Multiple preclinical and clinical studies have reported that EUG downregulates inflammatory mediators, including prostaglandins, cytokines, and chemokines, demonstrating efficacy in models of arthritis, colitis, and pulmonary inflammation [113]. Additionally, ortho-EUG was shown to reduce vascular permeability and leukocyte infiltration in an acetic acid-induced peritonitis model. It suppressed the expression of TNF-α and IL-1β by inhibiting the activation of NF-κB and p38 MAPK signaling pathways [114]. Furthermore, clove extract at 100 mg/well inhibited the production of IL-1β, IL-6, and IL-10, exerting anti-inflammatory effects both prior to and following LPS stimulation. While EUG did not significantly impact IL-1β levels, it did inhibit IL-6 and IL-10 production. Specifically, EUG effectively countered IL-6 production regardless of timing, and suppressed IL-10 when administered post-LPS exposure [115]. Zhang et al. demonstrated that α-eugenol glycoside (α-EG) exhibited potent anti-inflammatory activity in both acellular and cellular systems. Notably, its efficacy in non-cellular environments was enhanced by α-glucosidase, which is widely present in the cytoplasm. Moreover, α-EG showed superior anti-inflammatory effects compared to its parent compound, EUG, in cellular models [116]. A study conducted by Costa et al. demonstrated that the EUG derivative, bis-eugenol, exhibited significantly stronger antioxidant and anti-inflammatory properties compared to EUG itself. This enhanced efficacy is likely attributed to its unique chemical structure, which provides greater molecular stability and reduces the formation of phenoxy radicals. Mechanistically, bis-eugenol was shown to concurrently inhibit the TLR4/NF-κB signaling pathways while promoting the activation of NRF2 and the anti-inflammatory cytokine IL-10, indicating a dual mode of action involving both suppression of pro-inflammatory signaling and stimulation of endogenous protective mechanisms. These findings were supported by analyses of TNF-α expression, cell viability assays, and measurements of antioxidant enzyme activity [117]. A study by Baskaran et al. examined the hepatoprotective effects of EUG in a rat model of thioacetamide-induced liver injury. Pretreatment with EUG significantly mitigated hepatic damage by reducing cytochrome P450 2E1 (CYP2E1) activity, markers of lipid peroxidation and protein oxidation, as well as pro-inflammatory mediators. Additionally, EUG enhanced the overall antioxidant defense system. Single-cell gel electrophoresis (comet assay) demonstrated that EUG effectively prevented thioacetamide-induced DNA strand breaks. Furthermore, the upregulation of cyclooxygenase-2 (COX-2) gene expression triggered by thioacetamide exposure was reversed by EUG administration [118].

As summarized in Table 2, EUG and its derivatives exhibit diverse anti-inflammatory and antioxidant mechanisms, making them promising candidates for therapeutic applications. In models of acute lung injury, EUG reduced pro-inflammatory cytokines and oxidative stress markers while enhancing antioxidant enzyme activity. It has also shown efficacy in autoimmune conditions like rheumatoid arthritis by inhibiting cytokine secretion and reactive species formation. Molecular docking studies revealed strong COX-2 binding, and in vivo experiments with EUG and acetyleugenol confirmed their anti-inflammatory potential. Additionally, EUG and its isomers inhibit key inflammatory pathways such as NF-κB, p38 MAPK, and TLR4, and promote protective mechanisms like NRF2 activation and IL-10 production. Modified forms like bis-eugenol demonstrated superior stability and efficacy, while α-eugenol glycoside showed enhanced effects in cellular systems. Furthermore, eugenol has shown hepatoprotective effects by reducing CYP2E1 activity, lipid peroxidation, and DNA damage. Collectively, these findings underscore eugenol’s dual role in suppressing inflammation and oxidative damage across multiple biological systems.

### 4.2. Antibacterial

THY, CAR and EUG exhibit considerable antibacterial and antifungal activities. They have shown efficacy against a broad spectrum of bacteria, including different food-borne and medically important pathogens [37]. Research indicates that they exhibit antibacterial effects by reducing biofilm formation, hindering bacterial motility, inhibiting membrane-bound ATPases and efflux pumps, and disrupting the cell wall membrane [5,119]. Their antibacterial activity has been frequently investigated against *S. aureus*, *Salmonella*, *Shigella*, and *E. coli* [120,121,122,123]. Our recent review article highlighted the diverse antibacterial mechanisms of action of these compounds against different bacterial strains, as summarized in Table 3 [89].

## 5. Polymer-Based Wound Dressing Scaffolds Enhanced with Selected EO Components

### 5.1. Nanofibers

Nanofibers are highly effective as drug delivery systems [133]. The term “nanofiber” originates from the combination of “nano” meaning one-billionth, and “fiber,” which refers to a thin, elongated structure. As a result, nanofibers are extremely fine fibers with diameters typically ranging from 50 to 300 nanometers. These systems provide high therapeutic effectiveness even at low doses and are widely used in the pharmaceutical field for wound dressings and to improve topical drug delivery and skin penetration [134]. Nanofiber wound dressings offer several advantages, including high porosity, a large surface-area-to-volume ratio, and small pore size [135]. These nanofibrous scaffolds closely mimic the structure of the extracellular matrix, supporting epithelial cell proliferation and the formation of new tissue. Their nanoscale fiber diameter aids in initiating hemostasis in damaged tissues, enhances dermal drug delivery, improves fluid absorption and cell respiration, and allows efficient gas exchange, collectively contributing to the prevention of bacterial infection [136]. Numerous studies have reported the use of nanofiber materials loaded with EO components such as EUG, CAR, THY or menthol to enhance wound healing, highlighting their potential to improve therapeutic outcomes through antimicrobial, anti-inflammatory, and regenerative properties.

#### 5.1.1. Nanofibers Loaded with Eugenol

Noori et al. developed a bi-component wound dressing of PCL/Cs electrospun nanofibers and EUG nanogel to investigate its effects on tissue healing in vivo. The results showed that electrospun nanofibers had an average diameter of 228 nm with uniform and smooth morphology aligned randomly. EUG-loaded nanogel showed an average size distribution of 126 nm. EUG-loaded nanogel and nanogel + nanofiber groups significantly reduced wound surface area over 21 days. Histological evaluations showed that EUG-loaded nanogel and nanogel + nanofiber groups developed the full-thickness epidermis with the complete epithelium and stratum corneum, angiogenesis, and low macrophage infiltration in which predominantly mature collagen fibers were poorly and well organized, respectively. The combination of EUG-loaded nanogel+PCL/Cs nanofiber accelerated wound healing by reducing inflammation and edema along with enhancing angiogenesis, collagen synthesis, and re-epithelialization [137]. Loudifa et al. valorized two varieties of date palm mesh, *Washingtonia robusta* (S1) and *Phoenix Dactylifera* L. (S2) by extracting their fibrous cellulose structures for potential application in wound dressings. EUG, extracted from cloves, was successfully incorporated into the fibers, significantly enhancing their antibacterial activity. The cellulose–EUG composites displayed strong inhibition against *E. coli* (3.8 mm and 2.3 mm), *S. aureus* (4.00 mm and 2.05 mm), and *S. epidermidis* (2.7 mm and 1.6 mm) for S1 and S2, respectively. This combination of cellulose and EUG provides both structural support and antimicrobial properties, making these fiber dressings highly promising for wound care applications [138]. Sethuram et al. fabricated effective nanofibers by incorporating a EUG-based microemulsion (EUGME) and silver nanoparticles (AgNPs) into polyvinyl alcohol using electrospinning. Optimal conditions included 9% polymer concentration, 25 kV voltage, a 15 cm needle-to-collector distance, and a 0.5 mL/h flow rate. The resulting EUGME–AgNP nanofibers were compared to silver bandaid-based AgNP nanofibers (SBD–AgNP–NFs) for antibacterial effectiveness and biocompatibility. EUGME–AgNPs demonstrated superior antibacterial activity against *S. aureus* and showed controlled silver ion release in simulated wound fluid. In biological evaluations, EUGME–AgNPs displayed higher lymphocyte viability (69.81%) and lower red blood cell lysis (19.44%) compared to SBD–AgNPs. These findings suggest that EUGME–AgNP nanofibers offer effective antibacterial action and sustained silver release, making them promising candidates for clinical wound care [139]. Mouro et al. incorporated 5% EUG extracted from cloves into electrospun PCL/PVA/chitosan (CS) fiber mats using water-in-oil (W/O) and oil-in-water (O/W) emulsions. The resulting nanofibers, both with and without EUG, showed a highly porous structure mimicking native extracellular matrix (ECM) and exhibited moderate wettability, favorable for moisture retention and fibroblast attachment. EUG-loaded mats demonstrated antibacterial activity against *S. aureus* and *P. aeruginosa*. In vitro release studies showed an initial burst release within the first 8 h, followed by sustained release over several days, with the O/W emulsion offering better-controlled release than the W/O emulsion. Overall, the EUG-loaded electrospun mats were non-cytotoxic and demonstrated promising potential for enhanced wound healing applications [140]. Unalan et al. developed antibacterial electrospun nanofiber mats by combining poly(ε-caprolactone) (PCL) and gelatin (GEL) with clove essential oil (CEO), using glacial acetic acid as a safe solvent. The incorporation of CEO increased the fiber diameter from 241 ± 96 nm to 305 ± 82 nm and improved the mats’ wettability. FTIR and GC-MS confirmed the successful integration and content of CEO in the fibers. The CEO-loaded PCL-GEL mats showed no cytotoxicity toward normal human dermal fibroblast (NHDF) cells and exhibited antibacterial activity against *Staphylococcus aureus* and *Escherichia coli*, highlighting their potential for antibiotic-free wound healing [141]. Similarly, Hameed et al. formulated CEO-loaded nanofiber mats using chitosan and polyethylene oxide (PEO), achieving a fiber diameter of 154 ± 35 nm and high CEO encapsulation (87.6%) and loading efficiency (8.9%). These mats demonstrated excellent swelling (117%) and released 79% of CEO at pH 5.5. They also showed strong antibacterial activity against *S. aureus* and *E. coli*, were non-toxic to human fibroblasts, and displayed favorable wound-healing properties [142]. Studies on nanofibers loaded with EUG demonstrate their strong potential in wound healing applications (Table 4). Various approaches such as PCL/chitosan nanofibers combined with EUG nanogels, cellulose–EUG composites, EUG-based microemulsions with silver nanoparticles, and electrospun fiber mats with PCL, PVA, or chitosan consistently showed enhanced antibacterial activity against *S. aureus*, *E. coli*, and *P. aeruginosa*, as well as reduced inflammation, edema, and macrophage infiltration. These systems promoted angiogenesis, collagen synthesis, re-epithelialization, and tissue regeneration while remaining non-cytotoxic and biocompatible. Collectively, EUG-loaded nanofiber scaffolds provide both antimicrobial and structural support, making them promising candidates for advanced wound dressings.

#### 5.1.2. Nanofibers Loaded with Carvacrol/Thymol

Wang et al. developed electrospun nanofiber membranes composed of polyacrylonitrile/poly(ethylene oxide) (PAN/PEO) loaded with varying concentrations of CAR (CAR) for potential use in wound dressings. Using SEM and FTIR, they confirmed the morphology and chemical structure of the membranes. Antimicrobial tests showed that the PAN/PEO/CAR membranes exhibited concentration-dependent antibacterial activity. Further analysis using SEM and TEM revealed significant damage to Staphylococcus aureus biofilms and a reduction in bacterial count. In a full-thickness skin infection model, the membrane containing 5% CAR reduced wound exudate by day 2 and accelerated complete skin regeneration compared to the control. Gene pathway analysis indicated that the treatment downregulated genes related to S. aureus infection and its two-component regulatory system. Overall, the PAN/PEO/CAR nanofiber membranes demonstrated effective antimicrobial activity and enhanced wound healing, highlighting their potential for clinical wound care applications [143].

Güler et al. fabricated and analyzed polyvinylpyrrolidone (PVP) nanofibers loaded with various concentrations of CAR and lanolin using electrospinning. The additive concentrations ranged from 2.5 to 15 wt%, and the effects on solution properties such as viscosity, conductivity, and surface tension were evaluated. Results showed that increasing CAR:lanolin concentration led to higher viscosity and lower conductivity, while surface tension remained unchanged. Characterization using SEM, FT-IR, TGA, and DSC revealed that the nanofibers were smooth, fine, and uniform, with improved fiber quality at higher concentrations. FT-IR confirmed the successful incorporation of CAR and lanolin into the fibers. Thermal analysis indicated that the poor thermal stability of CAR and lanolin was enhanced when embedded in the PVP matrix. Given CAR’s antimicrobial effects and lanolin’s wound healing properties, these composite nanofiber mats show strong potential for biomedical applications, particularly as wound dressings [144].

In another study, THY was incorporated into polyamide nanofibers using the CO_2_ impregnation technique. The resulting THY-infused fibers demonstrated strong antibacterial activity against *E. coli*, *S. aureus*, and *C. albicans* [145]. Chen et al. developed porous THY-loaded CA fibrous membranes using a simple electrospinning method. Surface nanopores formed during fabrication enhanced the hydrophobicity of the membranes by trapping air. In vitro drug release studies showed that the porous membranes had a slower initial release and prolonged drug delivery compared to nonporous membranes, primarily governed by Fickian diffusion. Antibacterial results demonstrated that the porous THY-loaded CA fibrous membranes effectively inhibited *S. aureus* and *E. coli*, with bacterial survival rates of only 0.07% and 0.09%, respectively. Furthermore, the combination of the porous structure and controlled drug release promoted L929 cell proliferation, indicating improved cytocompatibility. Taken together, these findings suggest that porous THY-loaded CA fibrous membranes hold significant promise as novel wound healing materials [146]. García-Salinas et al. developed electrospun polycaprolactone (PCL) fibers containing approximately 14.92% *w*/*w* THY, with an average fiber diameter of around 299 nm. The mats exhibited suitable mechanical properties, including a tensile strength of 3.0 ± 0.5 MPa and an elongation at break of 74.4 ± 9.5%, making them promising candidates for wound dressing applications. In vivo experiments using SKH1 hairless mice demonstrated that the THY-loaded PCL fibers effectively inhibited the growth of *S. aureus* (ATCC 25923), with antibacterial efficacy comparable to that of chlorhexidine. Histological evaluations revealed minimal inflammation in the treated wounds. Overall, these results suggest that PCL-based wound dressings incorporating natural agents like THY can effectively prevent infection and support wound healing and tissue regeneration [147]. García-Salinas et al. developed a series of electrospun polycaprolactone (PCL) nanofiber patches infused with natural compounds from essential oils, including THY and tyrosol (TYR), aimed at reducing inflammation. Their study compared the effects of these compounds on activated macrophages when incorporated into the PCL matrix. Among the formulations, PCL-THY showed the most effective downregulation of pro-inflammatory genes associated with the NF-κB signaling pathway, outperforming both PCL-TYR and the combined PCL-TYR-THY patch. However, PCL-THY exhibited low cell adhesion, which could potentially limit wound adherence. Despite this, the findings suggest that THY-loaded PCL patches are promising candidates for wound dressings due to their antibacterial and anti-inflammatory properties, offering a potential alternative to conventional antibiotic-based materials [148].

In a study by Chen et al., THY was self-assembled with β-cyclodextrin (β-CD) to form a water-soluble inclusion complex (THY/β-CD) at a 1:1 molar ratio, as confirmed by ^1^H NMR analysis. This complex, known for its antibacterial properties, was then incorporated into CA fibrous membranes with a hierarchical structure via electrospinning. In vitro release studies showed that the CA/THY/β-CD membranes achieved a favorable sustained drug release profile. The membranes also demonstrated adequate mechanical strength suitable for biomedical use. Enhanced antibacterial activity was observed, attributed to the improved solubility and higher loading efficiency of THY within the β-CD complex. Furthermore, in vitro cell viability tests confirmed excellent cytocompatibility. Collectively, these findings highlight the strong potential of CA/THY/β-CD fibrous membranes as advanced wound healing materials [149]. Studies on nanofibers loaded with CAR and THY reveal significant antimicrobial and wound-healing potential (see Table 4). CAR-loaded electrospun nanofibers, including PAN/PEO and PVP-based systems, exhibited concentration-dependent antibacterial activity, biofilm disruption, and accelerated skin regeneration, while also enhancing the stability of CAR when embedded in polymers. THY-loaded nanofibers, developed using electrospinning, CO_2_ impregnation, and β-cyclodextrin inclusion complexes, demonstrated strong antibacterial effects against *S. aureus*, *E. coli*, and *C. albicans*, along with controlled drug release, cytocompatibility, and improved tissue regeneration. Overall, CAR- and THY-based nanofiber scaffolds combine antimicrobial and anti-inflammatory properties with structural support, underscoring their potential as advanced materials for wound care.

### 5.2. Hydrogels

Hydrogels are three-dimensional, hydrophilic polymer networks capable of absorbing substantial amounts of water or biological fluids, making them highly suitable for various biomedical applications. These include uses in soft contact lenses, tissue engineering scaffolds, controlled drug delivery systems, and wound dressings [150]. Hydrogels can be produced from either synthetic polymers or biopolymers. Those derived from natural, biodegradable polymers such as polysaccharides, polypeptides, and proteins offer several advantages over synthetic counterparts and have recently attracted significant attention. An important advantage is their porous macromolecular structure, which can be readily tailored to encapsulate various bioactive compounds and enable their controlled release [151,152].

Han et al. investigated polyurethane–gelatin (PG) hydrogels infused with EUG (0.5% and 1%) as wound dressings. In vivo studies showed that the EUG-loaded hydrogels accelerated diabetic wound healing by promoting angiogenesis, cell proliferation, tissue regeneration, and re-epithelialization, leading to enhanced antibacterial and angiogenic effects. In vitro, the hydrogels stimulated fibroblast (HFF-1) and endothelial cell (HUVEC) growth while exhibiting strong antibacterial activity. These findings demonstrate the promise of EUG-infused hydrogels for effective treatment of diabetic wound defects [153]. Khadim et al. developed chitosan–oxidized microcrystalline cellulose (CS.OMCC) hydrogels loaded with EUG, exhibiting strong antimicrobial, biocompatible, and angiogenic properties. Four hydrogel ratios were prepared via Schiff base (–C=N) linkages between chitosan and OMCC. The hydrogels showed a maximum swelling capacity of 1165% and a weight loss of 56.8% in degradation studies. Antibacterial tests revealed potent activity against *E. coli* and *S. aureus*, with the 4:1 CS.OMCC hydrogel containing 30% EUG producing the largest inhibition zone (17 mm). The materials supported fibroblast adhesion and migration, while in vivo CAM assays demonstrated increased blood vessel formation, indicating strong angiogenic potential. Elevated VEGF expression further confirmed this effect. Overall, EUG-loaded CS.OMCC hydrogels offer a promising strategy for combating bacterial infections and enhancing angiogenesis to accelerate wound healing [154]. Zhou et al. developed an injectable multifunctional hydrogel composed of laponite (LAP) and lactoferrin (LF), loaded with 2% EUG via a simple one-pot electrostatic assembly method. Designed to adapt to irregular wound shapes, the LAP/LF/EUG hydrogel provided sustained release of LF and EUG, effectively reducing oxidative stress and eliminating bacterial infections. In MRSA-infected wound models, the hydrogel demonstrated excellent injectability, strong antioxidant and hemostatic properties, and high cytocompatibility. It promoted cell migration, inhibited multidrug-resistant MRSA growth, and accelerated healing by enhancing angiogenesis, collagen deposition, and re-epithelialization. Overall, this hydrogel shows great potential as an advanced wound dressing for managing bacterial-infected wounds and promoting tissue regeneration [155]. Li et al. developed a bioactive carboxymethylcellulose hydrogel incorporating EUG–β-cyclodextrin inclusion complexes to enhance angiogenesis and reduce inflammation for improved diabetic wound healing. The hydrogel demonstrated strong antibacterial activity both in vitro and in vivo. In animal studies, it accelerated wound closure by suppressing LOX-1/NF-κB-induced endothelial cell dysfunction and promoting new blood vessel formation, highlighting its potential as an effective treatment for diabetic wounds [156]. Qiu et al. developed a degradable, pH-responsive hyaluronic acid hydrogel dressing for wound healing. Aldehyde-modified hyaluronic acid (AHA) was synthesized and combined with carboxymethyl chitosan (CMCS) via a Schiff base reaction to encapsulate EUG and oregano essential oils with antibacterial properties. The hydrogel exhibited marked pH sensitivity, with its disintegration mass at acidic pH (5.5) being four times greater than at neutral pH (7.2), leading to a substantial increase in EUG release (from 37.6% to 82.1%). Antibacterial assays demonstrated a fivefold improvement in biofilm removal efficiency, while in vivo tests confirmed significantly accelerated wound healing. With its potent anti-biofilm activity and targeted release capability, this hydrogel shows strong potential for managing bacteria-associated wounds [157].

Jiji et al. fabricated a THY-enriched bacterial cellulose hydrogel (BCT) via a simple absorption process, leveraging the high water-retention capacity of bacterial cellulose. The BCT hydrogel displayed strong antibacterial activity against burn-associated pathogens. THY incorporation had minimal effect on the water vapor transmission rate (WVTR), helping to maintain wound moisture. In vitro testing with NIH 3T3 cell lines showed high cell viability and enhanced fibroblast proliferation, while in vivo experiments demonstrated faster wound healing through improved wound closure and re-epithelialization. This pioneering study highlights the effective integration of THY into bacterial cellulose for burn wound dressings, suggesting its potential for clinical use in burn care and other biomedical applications [158]. Cui et al. developed a natural, carrier-free, self-assembled injectable hydrogel (THY@glycygel) as a wound dressing to address bleeding, bacterial infection, and promote skin healing. The system was prepared by loading THY into glycyrrhizin-based micelles (THY@glycymicelles), which enhanced THY’s water solubility. Through eco-friendly physical crosslinking, without chemical agents and pH adjustment, the micelles could reversibly transform into THY@glycygel and back without altering THY content or key physicochemical properties. Cryo-SEM imaging revealed a compact network formed by self-assembled glycyrrhizin. This THY@glycygel exhibited strong in vitro and in vivo antibacterial activity against MRSA, *S. aureus*, and *E. coli*, killing bacteria by disrupting their cell walls and membranes. In MRSA-infected wounds, it continuously suppressed bacterial growth, stimulated hair follicle formation, enhanced epidermal remodeling, boosted collagen deposition, reduced pro-inflammatory cytokines (IL-1β, IL-17A), and regulated wound-healing growth factors (VEGF, CD31, α-SMA). The hydrogel showed no toxicity to major organs or skin, highlighting its potential as a safe, natural, and effective treatment for bacterial-infected wounds and as a model for developing carrier-free injectable hydrogels [159]. Mohsen et al. developed THY-loaded cationic polymeric nanoparticles (CPNPs) to improve skin retention and wound-healing efficacy of THY. The nanoparticles showed entrapment efficiencies of 56.58–68.97%, particle sizes ranging from 36.30–99.41 nm, and a positive zeta potential, with sustained THY release for up to 24 h in vitro. Selected formulations (F5 and C2) were incorporated into methylcellulose-based hydrogels (GF5 and GC2). In vivo studies demonstrated that GF5 and GC2 achieved 3.3- and 3.6-fold higher skin retention than free THY, respectively. In vitro scratch assays showed significantly faster wound closure at 24 h: 58.09% for GF5 and 57.45% for GC2. In a murine MRSA-infected skin model, all hydrogels (free THY, GF5, GC2) reduced bacterial counts in skin lesions, but only GF5 and GC2 prevented bacterial spread to the spleen. These findings indicate that Eudragit RS30D-based nanoparticle hydrogels are a promising delivery platform for enhancing THY’s skin retention, antibacterial activity, and wound-healing potential [160]. Moradi et al. reported the development of pH-sensitive chitosan/polyvinyl alcohol (CS/PVA) hydrogels incorporating thyme oil inclusion complexes with methyl-β-, hydroxypropyl-β-, and γ-cyclodextrin (TM-CD-ICs) using a controlled, biocompatible, and cost-effective freeze–thaw method. Structural and morphological analyses via FTIR, optical microscopy, and SEM confirmed the stability of the hydrogels, which exhibited good mechanical strength, high swelling ratios, and water vapor transmission rates within the ideal range for wound dressings. Encapsulation and release profiles of TM-CD-ICs were monitored using UV spectroscopy, with drug release kinetics evaluated by various mathematical models. Hydrogels containing TM-CD-ICs demonstrated slower and more controlled release compared to non-encapsulated systems. Antibacterial assays against both Gram-positive and Gram-negative bacteria revealed notable antimicrobial activity, particularly for TM-γCD-ICs-loaded hydrogels. Biocompatibility assessments showed that TM-γCD-ICs hydrogels supported higher cell viability (MTT assay) and better cell attachment compared to TM-βCD-ICs formulations. These findings suggest that TM-γCD-ICs hydrogels possess optimal mechanical, antibacterial, and cytocompatibility properties, making them promising candidates for biomedical applications such as wound dressings to enhance healing and for use in controlled drug delivery systems [150]. Cuéllar-Gaona et al. developed chitosan-based hydrogels incorporating varying concentrations of CAR, characterized using FTIR, TGA, and SEM. Biocompatibility tests via direct hemolysis confirmed their safety for blood contact. Antimicrobial assays showed strong activity, with over 95% inhibition against *S. aureus* and over 90% against *E. coli*. The hydrogels also provided sustained CAR release for at least 48 h. These findings highlight chitosan/CAR hydrogels as promising candidates for biomedical applications, particularly in wound dressings and drug delivery, due to their combined biocompatibility, antimicrobial efficacy, and controlled-release properties [161]. Iqbal et al. developed sodium alginate (SA) hydrogel membranes incorporating oregano essential oil (OEO) rich in CAR and THY via the solvent casting method and assessed their physicochemical, antioxidant, and antibacterial properties. FTIR, SEM, swelling ratio, DSC, and encapsulation efficiency analyses (40.5%) confirmed molecular interactions between the components, a rough and porous surface morphology, and good thermal stability. Higher OEO content led to reduced swelling capacity but significantly improved antioxidant activity (DPPH assay) and antibacterial performance (disc diffusion) against tested microbes. Overall, SA/OEO hydrogel membranes demonstrate strong potential as wound-healing materials with dual antioxidant and antimicrobial functionalities [162]. Mahadevi et al. formulated herbal hydrogels using *Curcuma amada* rhizome extract combined with oregano oil (*Origanum vulgare*) and one of three polymers: carbopol 934, sodium alginate, or sodium carboxymethyl cellulose. The gels were evaluated for pH, viscosity, and spreadability, followed by in vivo excision wound healing and anti-inflammatory studies on male Wistar rats. Among the formulations, the carbopol 934-based gel demonstrated the best performance, showing high viscosity (65 × 10^3^ cPs), good spreadability (25 g × cm sec^−1^), complete in vitro release (100%), and significant wound contraction (350.97 ± 0.27 mm^2^), outperforming the positive control (neomycin sulfate). Additionally, the highest anti-inflammatory activity was observed in rats treated with 200 mg/kg of *C. amada* methanol extract compared to indomethacin. These findings suggest that a gel combining *C. amada* extract, oregano oil, and carbopol 934 is a promising candidate for treating wounds and inflammation [163]. In summary (see Table 5), hydrogels, due to their high water content, biocompatibility, and tunable porous structure, are widely explored as wound dressings capable of encapsulating and releasing bioactive compounds. Incorporation of EUG into hydrogels enhanced antibacterial, angiogenic, antioxidant, and tissue-regenerative properties, showing strong potential for diabetic and infected wound healing. Similarly, THY-based hydrogels, including bacterial cellulose and injectable systems, demonstrated potent antibacterial effects, improved collagen deposition, and accelerated re-epithelialization. CAR- and oregano oil–enriched hydrogels provided sustained antimicrobial action, antioxidant benefits, and favorable biocompatibility. Collectively, EO-loaded hydrogels combine controlled release, infection control, and enhanced tissue regeneration, making them promising candidates for advanced wound care applications.

### 5.3. Films

Films are transparent and adhesive polyurethane (PU)-based wound dressings that allow effective gaseous exchange between the wound and its surrounding environment [46]. The transparent nature of film dressings enables continuous observation of the wound healing process without the need for removal. They offer several advantages, including excellent mechanical strength, high flexibility, and elasticity, which allow them to conform easily to various shapes without requiring additional fixation. However, film dressings are unsuitable for wounds with heavy exudate, as their low porosity limits their ability to absorb large volumes of biological fluids [164]. Several studies have reported the effectiveness of films incorporating these selected EO components in promoting wound healing.

#### 5.3.1. THY/CAR Films

Ahmady et al. successfully developed and characterized a chitosan–gelatin (CS–GEL) composite film incorporated with THY-loaded alginate microparticles (THY-ALG MPs) for use as a wound dressing. The THY-loaded microparticles were prepared using the electrostatic droplet generation method, and SEM analysis confirmed their uniform and spherical morphology. Characterization of the CS–GEL/THY-ALG MPs composite film revealed a desirable swelling capacity and demonstrated a 2.5-fold enhancement in antibacterial activity against Gram-negative bacteria compared to the plain CS–GEL film. Furthermore, the THY release time from the composite film was prolonged by 3.5 and 1.7 times relative to THY-ALG MPs and the CS–GEL/THY film, respectively. DSC and FTIR analyses verified the structural composition of the composite film, while SEM imaging confirmed the successful incorporation of the microparticles into the polymer matrix. MTT assays indicated good cell viability and non-toxicity. Additionally, in vivo wound healing studies showed that the composite film significantly accelerated wound closure within 14 days compared to a commercial antibacterial dressing, and histological analysis revealed complete wound healing by day 21. These findings highlight the strong potential of the CS–GEL/THY-ALG MPs composite film as an effective wound dressing for clinical applications [165].

Acar et al. developed THY-loaded wound dressing films using polysaccharide polymers—gellan gum, carboxymethyl cellulose, and hyaluronic acid via the solvent casting method. The films were characterized by FTIR-ATR and SEM, confirming their structural integrity. They exhibited a high swelling capacity (~829% in 24 h), moderate water vapor transmission (~2376 g/m^2^/day), and hydrolytic degradation of ~55% over 21 days, all suitable for maintaining a moist wound environment. Incorporating THY improved the films’ flexibility, and increasing its concentration enhanced the cumulative release from 4.42 ± 0.40 mg/g to 6.25 ± 0.39 mg/g. In vitro studies using L929 fibroblast cells confirmed good biocompatibility, with the 5% THY film (GCH-5) significantly promoting cell migration and proliferation. These findings suggest that polysaccharide-based THY films are promising candidates for clinical wound dressing applications [166].

Pires et al. investigated the influence of poly(dimethylsiloxane) on the mechanical properties of chitosan–alginate (CA) polyelectrolyte complexes (PECs) for potential use as wound dressing biomaterials. Incorporating different amounts of poly(dimethylsiloxane) revealed that the formulation containing 0.1 g per gram of PEC (CAS10) exhibited the highest tensile strength, was non-hemolytic, promoted thrombus formation to help reduce bleeding, and remained stable under physiological conditions and simulated bathing environments. To enhance its wound healing potential, CAS10 was further loaded with THY and beta-carotene compounds with anesthetic, anti-inflammatory, and antioxidant properties using a supercritical CO_2_ impregnation/deposition (SSI/D) method. This approach achieved higher bioactive loadings (1.8 ± 0.2 μg/mg for THY and 1.3 ± 0.03 μg/mg for beta-carotene) at a depressurization rate of 10 bar/min compared to conventional impregnation. The strong interactions between the bioactives and the polymer matrix suggested a more sustained release profile, highlighting the potential of these PEC-based systems as advanced wound dressing materials [167].

Najafloo et al. developed a niosomal nanocarrier incorporated into fibroin films to enable localized and sustained delivery of THY, a plant-derived antimicrobial agent, for the prevention of implant-associated infections. THY-loaded niosomes were prepared using the thin-film hydration method, and their release profile from the films was monitored over 14 days. The films exhibited a sustained release pattern, reaching approximately 40% THY release within this period. MTT assays demonstrated that films containing THY, with or without niosomes, maintained significant viability of L929 fibroblast cells after 24 and 48 h. Moreover, the films showed strong antibacterial activity against both Gram-negative (*E. coli*, *P. aeruginosa*) and Gram-positive (*S. aureus*) bacteria. These findings suggest that niosomal thymol-loaded fibroin films hold great promise as controlled-release wound dressing systems for preventing implant-related infections [168].

Łopusiewicz et al. reported the development and bioactivity assessment of poly(butylene succinate) (PBS)-based films incorporating the natural bioactive compounds CAR and curcumin at concentrations of 0%, 0.1%, and 1% using the solvent casting method. The addition of CAR significantly enhanced the films’ antioxidant activity, with free radical scavenging reaching up to 91.47% (DPPH) and 99.21% (ABTS). The modified films also demonstrated potent antimicrobial properties, achieving reductions of 6 log for *E. coli*, 4 log for *S. aureus*, and 2 log for *Candida albicans* when containing 1% of both bioactives. Additionally, they exhibited strong antibiofilm activity, reducing biofilm formation by 8.22–87.91%, depending on the concentration used. These findings highlight the potential of CAR-modified PBS films as multifunctional biomaterials with antimicrobial, antioxidant, and antibiofilm properties, suitable for applications such as active packaging and wound dressing [169].

#### 5.3.2. EUG Films

Antunes et al. developed chitosan/polyvinyl alcohol (CS/PVA) blended films incorporated with EUG-containing EO (Cinnamon leaf (CO) and clove (CLO) oils) and evaluated their antibacterial activity against *S. aureus* and *P. aeruginosa* in infected environments. The films (30:70 *w*/*w*; 9 wt%) were fabricated using solvent casting and phase inversion techniques. Characterization confirmed successful polymer blending and essential oil encapsulation, with film thickness and swelling capacity increasing alongside EO content, especially at 10 wt%. UV–Vis analysis (250–320 cm^−1^) verified efficient oil incorporation, showing over fivefold higher CLO/CO loading in 10 wt% films compared to 1 wt%. Antimicrobial assays demonstrated that even unloaded CS films could eliminate *P. aeruginosa* within 1 h, while EO-loaded films exhibited significantly enhanced antibacterial performance within 2 h, particularly against *S. aureus*. This work provides proof of concept that CLO and CO can be effectively incorporated into CS/PVA films to deliver potent bactericidal effects, highlighting their potential for chronic wound treatment applications [170].

The studies reviewed in this section demonstrate that EO-incorporated films significantly enhance wound healing outcomes (Table 6). THY-loaded films showed increased antibacterial activity, sustained release, high swelling capacity, and promoted fibroblast proliferation and migration. CAR- and curcumin-modified PBS films exhibited strong antimicrobial, antioxidant, and antibiofilm properties. EUG-containing CS/PVA films effectively eliminated *P. aeruginosa* and *S. aureus*, with enhanced bioactive loading and swelling at higher EO concentrations. Overall, these films combine biocompatibility, controlled release, and potent antimicrobial effects, leading to accelerated wound closure and improved tissue regeneration.

Although hydrogels, nanofibers and films incorporating the selected EO components have been widely explored, reports on other biomaterials such as aerogels, cryogels, sponges, bandages, foams, wafers, patches, etc., remain scarce. This highlights an opportunity for future research to investigate their potential in wound healing applications.

## 6. Conclusions

Wound healing remains a complex and multifaceted biological process requiring targeted therapeutic strategies to promote effective tissue regeneration and prevent infection. This review highlights the promising potential of polymer-based scaffolds incorporating selected EO components such as CAR, THY, and EUG in enhancing wound healing outcomes. The synergistic integration of biocompatible polymers and bioactive EO constituents not only enhances the structural and mechanical properties of the scaffolds but also imparts antimicrobial, anti-inflammatory, and antioxidant effects that are crucial for efficient wound management. Various studies have demonstrated that these composite systems can mimic the extracellular matrix, promote cell proliferation and migration, and accelerate tissue regeneration while reducing infection risks. Despite the encouraging in vitro and in vivo findings, challenges such as controlled release, stability of EO components, and standardization of formulations still need to be addressed. Future research should focus on optimizing scaffold design, exploring novel polymer-EO combinations, and conducting comprehensive clinical studies to validate their safety and efficacy. Overall, polymer-based scaffolds loaded with selected EO components hold significant potential as next-generation wound dressings aimed at enhancing healing outcomes and improving patient care.

## Figures and Tables

**Table 1:** Anti-Inflammatory Mechanisms and Therapeutic Effects of CAR and THY.

Compound	Mechanism of Action	Therapeutic Effect/Context	Reference
CAR & THY	Modulation of MAPK, NF-κB, JAK/STAT, and arachidonic acid pathways	Reduction of pro-inflammatory cytokines and mediators	[7]
CAR	Inhibits edema formation and leukocyte infiltration	Treatment of inflammatory skin conditions; promotes wound healing	[103]
CAR & THY	Reduced edema (both), but only CAR inhibited leukocyte migration; THY induced chemoattractant response	Local anti-inflammatory effects in edema and pleurisy models	[29]
CAR & THY	Calcium channel blockade	Anti-inflammatory and uterine relaxant effects; potential tocolytics	[104]
CAR	↓ IL-1β and TNF-α (mRNA and protein); ↓ c-Fos, NFAT-1/2, c-Jun; ↑ phospho-p38; ↓ phospho-STAT3 and phospho-SAPK/JNK	Broad anti-inflammatory signaling modulation	[105]
THY	↓ IL-1β; ↓ c-Fos, NFAT-1/2; ↓ phospho-STAT3 and phospho-SAPK/JNK	Targeted anti-inflammatory effect, but less extensive than carvacrol	[105]
CAR & THY	↓ COX-1, COX-2, 5-LOX enzyme expression	Synergistic anti-inflammatory and cardioprotective effects	[106,107]
CAR/THY derivatives	Inhibited LPS-induced IL-1β in BV2 microglial cells (IC_50_ ≈ 8.33 μM); ↓ IL-1β in AD mouse model	Potent anti-inflammatory and neuroprotective effects; improved cognition	[108]

Note: “↑” indicates upregulation or increase; “↓” indicates downregulation or decrease.

**Table 2:** Summary of Eugenol’s Mechanisms of Action and Therapeutic Effects.

Mechanism of Action	Therapeutic Context/Effect	Reference
Suppression of TNF-α, IL-1β, IL-6 release; inhibition of NADPH oxidase; enhancement of antioxidant enzymes (SOD, catalase, GPx)	Anti-inflammatory and antioxidant effects in LPS-induced acute lung injury	[110]
Inhibition of cytokine secretion from PBMCs; reduction of ROS/RNS production	Anti-inflammatory and antioxidant activity in rheumatoid arthritis models	[111]
Favorable COX-2 binding affinity (eugenol and acetyleugenol); good ADMET profile	In silico drug-likeness and anti-inflammatory drug development	[112]
Downregulation of prostaglandins, cytokines, and chemokines	Prevention of cardiovascular and inflammatory diseases	[113]
Inhibition of NF-κB and p38 MAPK signaling; reduced TNF-α and IL-1β expression	Anti-inflammatory activity in peritonitis model	[114]
Inhibition of IL-6 and IL-10 (but not IL-1β) production in LPS-stimulated cells	Immune modulation and anti-inflammatory action	[115]
α-Eugenol glycoside (α-EG) showed enhanced anti-inflammatory activity, potentiated by α-glucosidase	Superior cellular anti-inflammatory effect compared to parent eugenol	[116]
Bis-eugenol inhibited TLR4/NF-κB and activated NRF2 and IL-10 pathways	Dual pro-resolution and antioxidant action; enhanced efficacy vs. eugenol	[117]
Reduction of CYP2E1 activity, lipid/protein oxidation, DNA strand breaks; reversal of COX-2 upregulation	Hepatoprotective and anti-inflammatory effects in thioacetamide liver injury	[118]

**Table 3:** Antibacterial mechanisms of action of THY, CAR and EUG.

	Antibacterial		
EO Components	Mechanism of Action	Sensitive Strains	Reference
THY	Disrupts cell membrane integrity, causing leakage of intracellular materials and eventual bacterial cell death.	*Aeromonas hydrophila*	[124]
	Penetrates bacterial cells, binds to the DNA minor groove, and destabilizes the secondary structure of DNA.	*S. aureus*	[125]
	Damages cell wall and membrane, leading to leakage of intracellular contents; interferes with energy metabolism, membrane transport, and DNA processes; inhibits binary division, nutrient uptake, metal ion transport, nucleotide biosynthesis, DNA repair, and transcriptional pathways	*S. iniae*	[126,127]
	Causes irreversible membrane damage, induces nucleic acid leakage, and generates reactive oxygen species (ROS), leading to DNA damage through intercalation.	*Pseudomonas aeruginosa*	[127]
CAR	Causes growth inhibition by damaging cell membranes, increasing permeability, and disrupting cell walls in both Gram-positive and Gram-negative bacteria.	*S. aureus*, *S. epidermidis*, *S. pneumoniae*, *E. coli*, *K. pneumoniae*, *Proteus mirabilis*, *Enterobacter* spp., *Serratia* spp.	[128]
EUG	Disrupts cell membrane integrity via oxidative stress, causing leakage of intracellular components; also effective in biofilm removal.	*Vibrio vulnificus*	[129]
	Reduces superoxide dismutase (SOD) activity, leading to ROS accumulation, oxidative membrane damage, ATP leakage, and altered membrane potential.	*Shigella flexneri*	[130]
	Inhibits biofilm formation by disrupting cell-to-cell interactions; detaches established biofilms, reduces viable cells, increases nucleic acid and protein leakage, decreases metabolic activity and extracellular polymeric substance (EPS) production, and reduces hydrophobicity, motility, and virulence.	*V. parahaemolyticus*	[131]
	Elevates intracellular ROS levels, decreases ATP concentration, induces membrane hyperpolarization, reduces membrane integrity, and alters cell morphology.	*S. sonnei*	[132]

**Table 4:** Summary of Nanofiber-Based Wound Dressings Loaded with EUG, CAR, and THY Composition, Key Findings, and Therapeutic Potential.

Nanofiber Composition	Active Compound(s)	Key Findings/Properties	Applications/Potential	Reference
PCL/Chitosan electrospun nanofibers + EUG nanogel	EUG	Nanofibers ~228 nm; EUG nanogel ~126 nm; reduced inflammation & edema; enhanced angiogenesis, collagen synthesis, and re-epithelialization	Bi-component wound dressing; improved tissue healing	[137]
Cellulose fibers from date palm mesh + EUG	EUG	Strong antibacterial activity (*E. coli*, *S. aureus*, *S. epidermidis*); provided structural support & antimicrobial effect	Antibacterial wound dressings	[138]
PVA nanofibers + EUG-based microemulsion + AgNPs	EUG + Silver NPs	Superior antibacterial effect (*S. aureus*); controlled Ag release; good lymphocyte viability; low RBC lysis	Clinical wound care; antibacterial dressings	[139]
PCL/PVA/Chitosan nanofibers with 5% EUG	EUG	Porous ECM-like structure; antibacterial vs. *S. aureus*, *P. aeruginosa*; burst + sustained release; non-cytotoxic	Enhanced wound healing	[140]
PCL/Gelatin nanofibers + Clove Essential Oil (CEO)	Clove EO (EUGl-rich)	Increased fiber diameter & wettability; antibacterial vs. *S. aureus*, *E. coli*; non-toxic to fibroblasts	Antibiotic-free wound healing	[141]
Chitosan/PEO nanofibers + CEO	Clove EO	High CEO encapsulation; excellent swelling; 79% release at pH 5.5; antibacterial vs. *S. aureus*, *E. coli*; non-toxic	Wound healing applications	[142]
PAN/PEO nanofibers + CAR	CAR	Concentration-dependent antibacterial activity; damaged *S. aureus* biofilms; reduced wound exudate; accelerated regeneration	Antimicrobial wound dressings	[143]
PVP nanofibers + CAR + Lanolin	CAR + Lanolin	Improved fiber quality & thermal stability; antimicrobial & wound healing properties	Biomedical wound dressings	[144]
Polyamide nanofibers + THY	THY	Strong antibacterial effect (*E. coli*, *S. aureus*, C. albicans)	Antibacterial wound dressings	[145]
Porous Cellulose Acetate nanofibers + THY	THY	Controlled release; antibacterial vs. *S. aureus*, *E. coli*; promoted cell proliferation	Novel wound healing materials	[146]
PCL nanofibers + THY	THY	Good mechanical properties; inhibited *S. aureus* in vivo; minimal inflammation; comparable to chlorhexidine	Infection prevention & tissue regeneration	[147]
PCL nanofiber patches + THY + Tyrosol	THY, Tyrosol	Thymol downregulated NF-κB inflammatory genes; low cell adhesion; antibacterial & anti-inflammatory	Wound dressings with anti-inflammatory activity	[148]
Cellulose Acetate nanofibers + THY/β-Cyclodextrin complex	THY (complexed)	Sustained release; improved solubility/loading; strong antibacterial activity; excellent cytocompatibility	Advanced wound healing materials	[149]

**Table 5:** Hydrogels Grouped by Active Compounds for Wound Healing.

Nanofiber Composition	Active Compound(s)	Key Findings/Properties	Applications/Potential	References
Eugenol-Based Hydrogels				
Polyurethane–gelatin (PG) hydrogel	EUG	Accelerated diabetic wound healing; promoted angiogenesis, proliferation, tissue regeneration; strong antibacterial activity	Diabetic wound treatment	[153]
Chitosan–oxidized microcrystalline cellulose (CS.OMCC) hydrogel	EUG	High swelling (1165%), biodegradable; antibacterial (*E. coli*, *S. aureus*); ↑ VEGF expression; angiogenic	Wound healing & infection control	[154]
Injectable LAP/LF hydrogel	EUG	Sustained release; antioxidant, antibacterial (MRSA); angiogenesis, collagen deposition; excellent injectability	MRSA-infected wound healing	[155]
Carboxymethylcellulose hydrogel	EUG–β-cyclodextrin complex	Antibacterial, angiogenesis; ↓ inflammation via LOX-1/NF-κB suppression	Diabetic wound healing	[156]
Hyaluronic acid (AHA) + Carboxymethyl chitosan hydrogel	EUG + Oregano essential oil	pH-responsive release (↑ 82.1% at pH 5.5); anti-biofilm; accelerated wound healing	Bacteria-associated wounds	[157]
Thymol-Based Hydrogels				
Bacterial cellulose hydrogel (BCT)	THY	High water retention; antibacterial (burn pathogens); fibroblast proliferation; enhanced re-epithelialization	Burn wound dressing	[158]
Injectable THY@glycygel (glycyrrhizin micelles)	THY	Carrier-free, reversible sol–gel; antibacterial (MRSA, *S. aureus*, *E. coli*); angiogenesis, collagen deposition; ↓ inflammatory cytokines	Bacterial-infected wound treatment	[159]
Cationic polymeric nanoparticles (CPNPs) in methylcellulose hydrogel	THY	Sustained release; ↑ skin retention (3.3–3.6 fold); faster wound closure; prevented bacterial spread	MRSA-infected wound healing	[160]
Chitosan/PVA hydrogel (freeze–thaw)	Thyme oil CD inclusion complexes (TM-β, HP-β, γ)	pH-sensitive, controlled release; antimicrobial (Gram +/–); biocompatible; ↑ cell viability	Wound dressings, controlled drug delivery	[150]
Carvacrol-Based Hydrogels				
Chitosan hydrogel	CAR	Sustained release (48 h); antimicrobial (>95% *S. aureus*, >90% *E. coli*); hemocompatible	Wound dressings & drug delivery	[161]
Sodium alginate hydrogel membrane	*Oregano* essential oil (CAR & THY)	Porous, thermally stable; antioxidant activity; antibacterial; swelling decreased with higher EO	Wound healing membranes	[162]
Other EO-Based/Herbal Hydrogels				
Herbal hydrogel (Carbopol 934, SA, NaCMC)	*C. amada* extract + *Oregano* oil	Carbopol 934 gel best: high viscosity, full drug release, strong wound contraction, anti-inflammatory activity	Wound healing & anti-inflammatory treatment	[163]

Note: “↑” indicates upregulation or increase; “↓” indicates downregulation or decrease.

**Table 6:** Summary of studies on selected EO components–incorporated film-based wound dressings, their compositions, bioactive agents, key properties, and potential applications.

Film Composition	Active Compound(s)	Key Findings/Properties	Applications/Potential	References
CS–gelatin composite film with thymol-loaded alginate microparticles	THY	2.5× higher antibacterial activity vs. Gram-negative bacteria; prolonged thymol release (3.5× vs. MPs, 1.7× vs. CS–GEL film); non-toxic with good cell viability; accelerated wound closure in 14 days and complete healing by day 21	Effective wound dressing with enhanced antibacterial and healing properties	[165]
Polysaccharide-based films (gellan gum, CMC, hyaluronic acid)	THY	High swelling (~829%/24 h), moderate WVTR (~2376 g/m^2^/day), ~55% degradation over 21 days; improved flexibility and release (4.42–6.25 mg/g); biocompatible and promoted fibroblast migration	Promising polysaccharide-based wound dressing	[166]
Chitosan–alginate PECs with poly(dimethylsiloxane) (CAS10)	THY & β-carotene	High tensile strength, non-hemolytic, thrombus formation, stable under physiological conditions; high bioactive loading (1.8 μg/mg thymol, 1.3 μg/mg β-carotene); sustained release profile	Advanced PEC-based wound dressing with anesthetic, anti-inflammatory, and antioxidant properties	[167]
Fibroin films with niosomal nanocarriers	THY	Sustained release (~40% over 14 days); maintained L929 fibroblast viability; strong antibacterial activity against *E. coli*, *P. aeruginosa*, and *S. aureus*	Controlled-release system for preventing implant-related infections	[168]
PBS-based films	CAR & Curcumin	Antioxidant activity (DPPH 91.47%, ABTS 99.21%); antimicrobial (6 log *E. coli*, 4 log *S. aureus*, 2 log *C. albicans*); antibiofilm (8.22–87.91%)	Multifunctional biomaterial for wound dressing and active packaging	[169]
CS/PVA blended films (30:70 *w*/*w*)	EUG-containing oils (clove & cinnamon leaf)	Increased thickness and swelling with 10 wt% EO; >5× higher EO loading vs. 1 wt%; unloaded CS eliminated *P. aeruginosa* in 1 h; EO-loaded films enhanced antibacterial action within 2 h, especially against *S. aureus*	Promising film for chronic wound treatment	[170]

## Data Availability

Not applicable.
